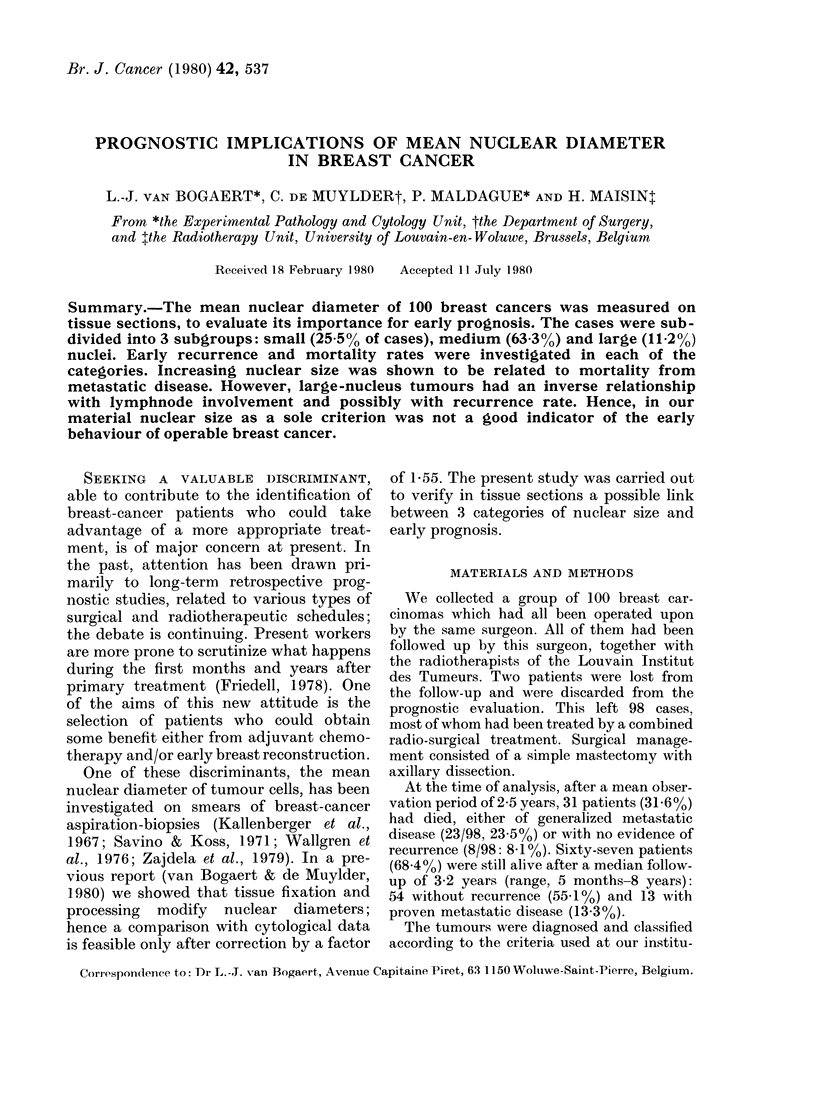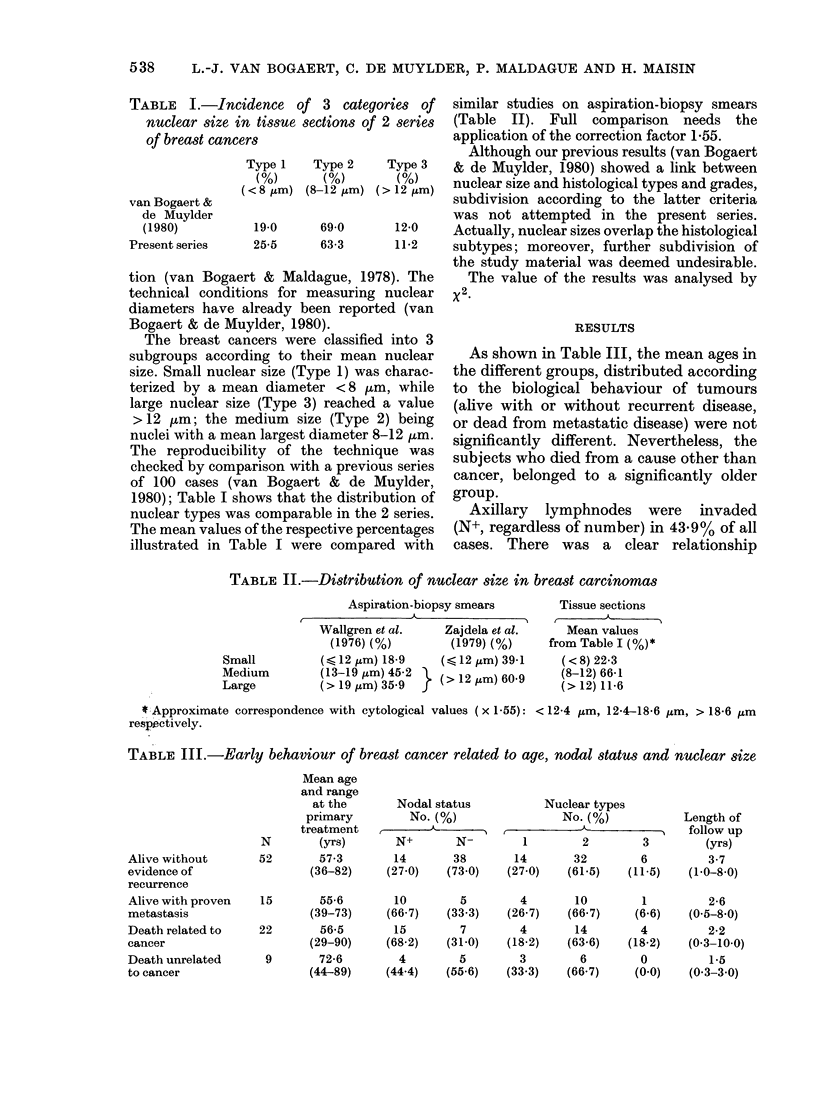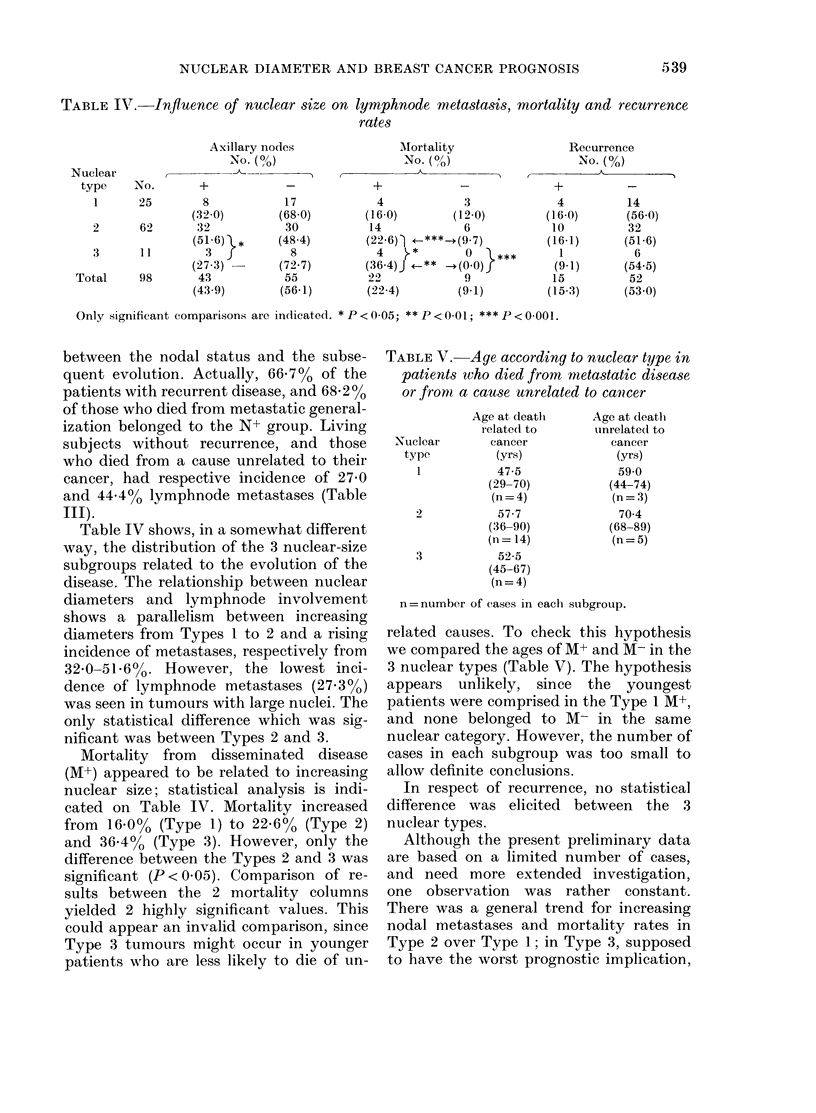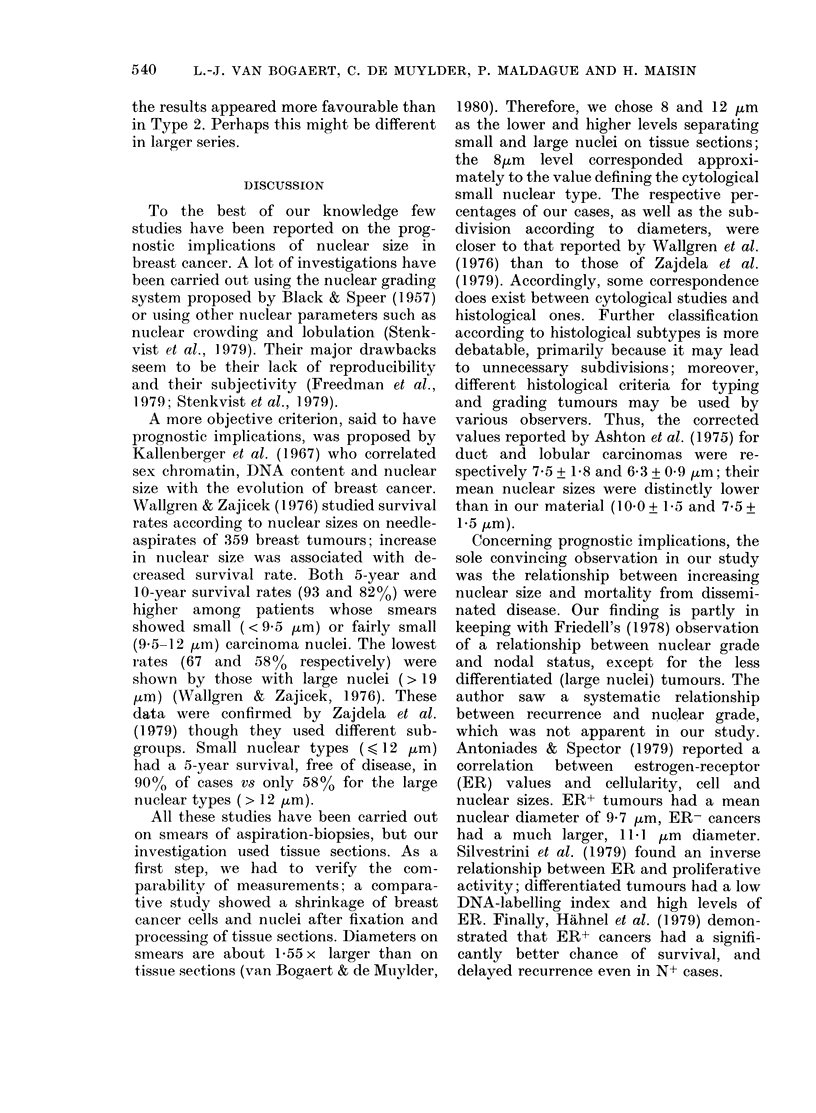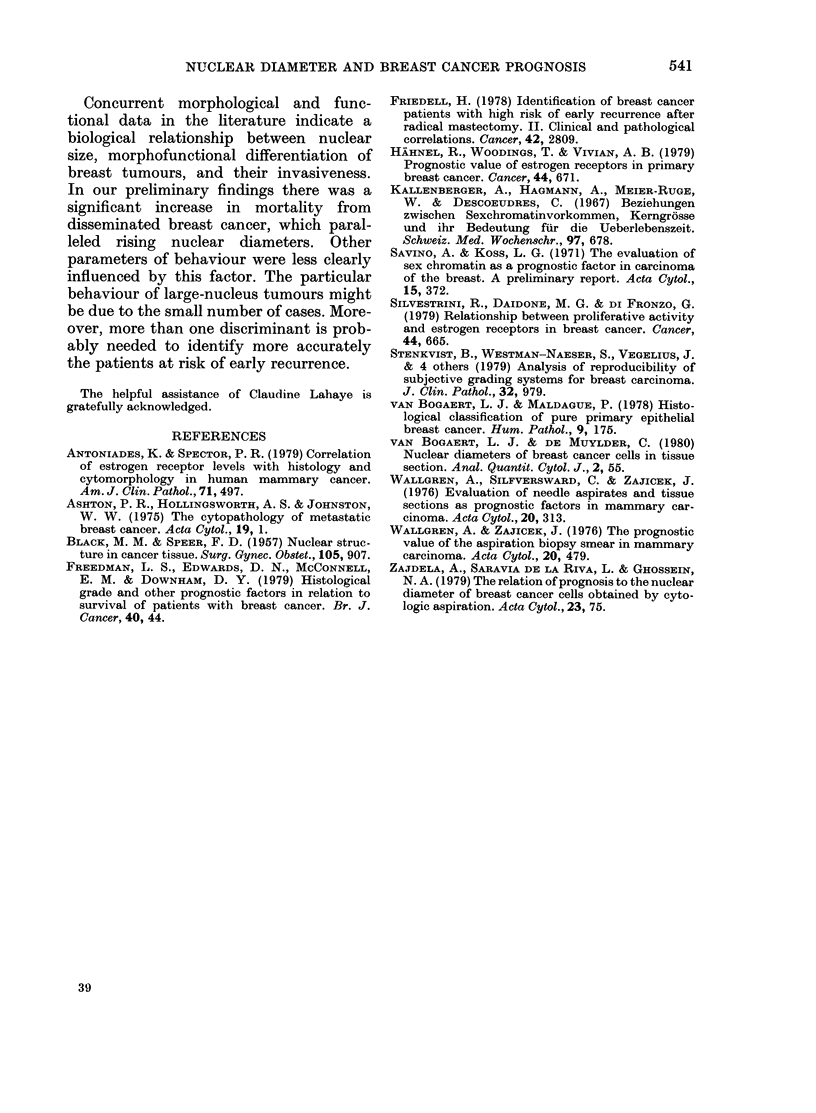# Prognostic implications of mean nuclear diameter in breast cancer.

**DOI:** 10.1038/bjc.1980.277

**Published:** 1980-10

**Authors:** L. J. van Bogaert, C. de Muylder, P. Maldague, H. Maisin

## Abstract

The mean nuclear diameter of 100 breast cancers was measured on tissue sections, to evaluate its importance for early prognosis. The cases were subdivided into 3 subgroups: small (25.5% of cases), medium (63.3%) and large (11.2%) nuclei. Early recurrence and mortality rates were investigated in each of the categories. Increasing nuclear size was shown to be related to mortality from metastatic disease. However, large-nucleus tumours had an inverse relationship with lymphnode involvement and possibly with recurrence rate. Hence, in our material nuclear size as a sole criterion was not a good indicator of the early behaviour of operable breast cancer.


*Br. J. Cancer* (1980) **42**, 537

### PROGNOSTIC IMPLICATIONS OF MEAN NUCLEAR DIAMETER IN BREAST CANCER

L.-J. VAN BOGAERT*, C. DE MUYLDER†, P. MALDAGUE* AND H. MAISIN‡

*From *the Experimental Pathology and Cytology Unit, †the Department of Surgery, and ‡the Radiotherapy Unit, University of Louvain-en-Woluwe, Brussels, Belgium*

Received 18 February 1980 Accepted 11 July 1980

**Summary.—The mean nuclear diameter of 100 breast cancers was measured on tissue sections, to evaluate its importance for early prognosis. The cases were subdivided into 3 subgroups: small (25·5% of cases), medium (63·3%) and large (11·2%) nuclei. Early recurrence and mortality rates were investigated in each of the categories. Increasing nuclear size was shown to be related to mortality from metastatic disease. However, large-nucleus tumours had an inverse relationship with lymphnode involvement and possibly with recurrence rate. Hence, in our material nuclear size as a sole criterion was not a good indicator of the early behaviour of operable breast cancer.**

SEEKING A VALUABLE DISCRIMINANT, able to contribute to the identification of breast-cancer patients who could take advantage of a more appropriate treatment, is of major concern at present. In the past, attention has been drawn primarily to long-term retrospective prognostic studies, related to various types of surgical and radiotherapeutic schedules; the debate is continuing. Present workers are more prone to scrutinize what happens during the first months and years after primary treatment (Friedell, 1978). One of the aims of this new attitude is the selection of patients who could obtain some benefit either from adjuvant chemotherapy and/or early breast reconstruction.

One of these discriminants, the mean nuclear diameter of tumour cells, has been investigated on smears of breast-cancer aspiration-biopsies (Kallenberger *et al.*, 1967; Savino & Koss, 1971; Wallgren *et al.*, 1976; Zajdela *et al.*, 1979). In a previous report (van Bogaert & de Muylder, 1980) we showed that tissue fixation and processing modify nuclear diameters; hence a comparison with cytological data is feasible only after correction by a factor of 1·55. The present study was carried out to verify in tissue sections a possible link between 3 categories of nuclear size and early prognosis.

### MATERIALS AND METHODS

We collected a group of 100 breast carcinomas which had all been operated upon by the same surgeon. All of them had been followed up by this surgeon, together with the radiotherapists of the Louvain Institut des Tumeurs. Two patients were lost from the follow-up and were discarded from the prognostic evaluation. This left 98 cases, most of whom had been treated by a combined radio-surgical treatment. Surgical management consisted of a simple mastectomy with axillary dissection.

At the time of analysis, after a mean observation period of 2·5 years, 31 patients (31·6%) had died, either of generalized metastatic disease (23/98, 23·5%) or with no evidence of recurrence (8/98: 8·1%). Sixty-seven patients (68·4%) were still alive after a median follow-up of 3·2 years (range, 5 months–8 years): 54 without recurrence (55·1%) and 13 with proven metastatic disease (13·3%).

The tumours were diagnosed and classified according to the criteria used at our institu-

Correspondence to: Dr L.-J. van Bogaert, Avenue Capitaine Piret, 63 1150 Woluwe-Saint-Pierre, Belgium.

TABLE I.—*Incidence of 3 categories of nuclear size in tissue sections of 2 series of breast cancers*

| | Type 1 (%) (< 8 μm) | Type 2 (%) (8–12 μm) | Type 3 (%) (> 12 μm) |
|---|---|---|---|
| van Bogaert & de Muylder (1980) | 19·0 | 69·0 | 12·0 |
| Present series | 25·5 | 63·3 | 11·2 |

tion (van Bogaert & Maldague, 1978). The technical conditions for measuring nuclear diameters have already been reported (van Bogaert & de Muylder, 1980).

The breast cancers were classified into 3 subgroups according to their mean nuclear size. Small nuclear size (Type 1) was characterized by a mean diameter <8 μm, while large nuclear size (Type 3) reached a value >12 μm; the medium size (Type 2) being nuclei with a mean largest diameter 8–12 μm. The reproducibility of the technique was checked by comparison with a previous series of 100 cases (van Bogaert & de Muylder, 1980); Table I shows that the distribution of nuclear types was comparable in the 2 series. The mean values of the respective percentages illustrated in Table I were compared with similar studies on aspiration-biopsy smears (Table II). Full comparison needs the application of the correction factor 1·55.

Although our previous results (van Bogaert & de Muylder, 1980) showed a link between nuclear size and histological types and grades, subdivision according to the latter criteria was not attempted in the present series. Actually, nuclear sizes overlap the histological subtypes; moreover, further subdivision of the study material was deemed undesirable.

The value of the results was analysed by $\chi^2$.

### RESULTS

As shown in Table III, the mean ages in the different groups, distributed according to the biological behaviour of tumours (alive with or without recurrent disease, or dead from metastatic disease) were not significantly different. Nevertheless, the subjects who died from a cause other than cancer, belonged to a significantly older group.

Axillary lymphnodes were invaded ($N^+$, regardless of number) in 43·9% of all cases. There was a clear relationship

TABLE II.—*Distribution of nuclear size in breast carcinomas*

| | Aspiration-biopsy smears | | Tissue sections |
|---|---|---|---|
| | Wallgren *et al.* (1976) (%) | Zajdela *et al.* (1979) (%) | Mean values from Table I (%)* |
| Small | (≤12 μm) 18·9 | (≤12 μm) 39·1 | (<8) 22·3 |
| Medium | (13–19 μm) 45·2 | (>12 μm) 60·9 | (8–12) 66·1 |
| Large | (>19 μm) 35·9 | | (>12) 11·6 |

* Approximate correspondence with cytological values (×1·55): <12·4 μm, 12·4–18·6 μm, >18·6 μm respectively.

TABLE III.—*Early behaviour of breast cancer related to age, nodal status and nuclear size*

| | N | Mean age and range at the primary treatment (yrs) | Nodal status No. (%) | | Nuclear types No. (%) | | | Length of follow up (yrs) |
|---|---|---|---|---|---|---|---|---|
| | | | $N^+$ | $N^-$ | 1 | 2 | 3 | |
| Alive without evidence of recurrence | 52 | 57·3 (36–82) | 14 (27·0) | 38 (73·0) | 14 (27·0) | 32 (61·5) | 6 (11·5) | 3·7 (1·0–8·0) |
| Alive with proven metastasis | 15 | 55·6 (39–73) | 10 (66·7) | 5 (33·3) | 4 (26·7) | 10 (66·7) | 1 (6·6) | 2·6 (0·5–8·0) |
| Death related to cancer | 22 | 56·5 (29–90) | 15 (68·2) | 7 (31·0) | 4 (18·2) | 14 (63·6) | 4 (18·2) | 2·2 (0·3–10·0) |
| Death unrelated to cancer | 9 | 72·6 (44–89) | 4 (44·4) | 5 (55·6) | 3 (33·3) | 6 (66·7) | 0 (0·0) | 1·5 (0·3–3·0) |

TABLE IV.—*Influence of nuclear size on lymphnode metastasis, mortality and recurrence rates*

| Nuclear type | No. | Axillary nodes No. (%) + | Axillary nodes No. (%) − | Mortality No. (%) + | Mortality No. (%) − | Recurrence No. (%) + | Recurrence No. (%) − |
|---|---|---|---|---|---|---|---|
| 1 | 25 | 8 (32·0) | 17 (68·0) | 4 (16·0) | 3 (12·0) | 4 (16·0) | 14 (56·0) |
| 2 | 62 | 32 (51·6) } * | 30 (48·4) | 14 (22·6) } * ←***→ | 6 (9·7) } *** | 10 (16·1) | 32 (51·6) |
| 3 | 11 | 3 (27·3) | 8 (72·7) | 4 (36·4) ←** → | 0 (0·0) | 1 (9·1) | 6 (54·5) |
| Total | 98 | 43 (43·9) | 55 (56·1) | 22 (22·4) | 9 (9·1) | 15 (15·3) | 52 (53·0) |

Only significant comparisons are indicated. * $P < 0{\cdot}05$; ** $P < 0{\cdot}01$; *** $P < 0{\cdot}001$.

between the nodal status and the subsequent evolution. Actually, 66·7% of the patients with recurrent disease, and 68·2% of those who died from metastatic generalization belonged to the $N^+$ group. Living subjects without recurrence, and those who died from a cause unrelated to their cancer, had respective incidence of 27·0 and 44·4% lymphnode metastases (Table III).

Table IV shows, in a somewhat different way, the distribution of the 3 nuclear-size subgroups related to the evolution of the disease. The relationship between nuclear diameters and lymphnode involvement shows a parallelism between increasing diameters from Types 1 to 2 and a rising incidence of metastases, respectively from 32·0–51·6%. However, the lowest incidence of lymphnode metastases (27·3%) was seen in tumours with large nuclei. The only statistical difference which was significant was between Types 2 and 3.

Mortality from disseminated disease ($M^+$) appeared to be related to increasing nuclear size; statistical analysis is indicated on Table IV. Mortality increased from 16·0% (Type 1) to 22·6% (Type 2) and 36·4% (Type 3). However, only the difference between the Types 2 and 3 was significant ($P < 0{\cdot}05$). Comparison of results between the 2 mortality columns yielded 2 highly significant values. This could appear an invalid comparison, since Type 3 tumours might occur in younger patients who are less likely to die of unrelated causes. To check this hypothesis we compared the ages of $M^+$ and $M^-$ in the 3 nuclear types (Table V). The hypothesis appears unlikely, since the youngest patients were comprised in the Type 1 $M^+$, and none belonged to $M^-$ in the same nuclear category. However, the number of cases in each subgroup was too small to allow definite conclusions.

TABLE V.—*Age according to nuclear type in patients who died from metastatic disease or from a cause unrelated to cancer*

| Nuclear type | Age at death related to cancer (yrs) | Age at death unrelated to cancer (yrs) |
|---|---|---|
| 1 | 47·5 (29–70) (n=4) | 59·0 (44–74) (n=3) |
| 2 | 57·7 (36–90) (n=14) | 70·4 (68–89) (n=5) |
| 3 | 52·5 (45–67) (n=4) | |

n = number of cases in each subgroup.

In respect of recurrence, no statistical difference was elicited between the 3 nuclear types.

Although the present preliminary data are based on a limited number of cases, and need more extended investigation, one observation was rather constant. There was a general trend for increasing nodal metastases and mortality rates in Type 2 over Type 1; in Type 3, supposed to have the worst prognostic implication, the results appeared more favourable than in Type 2. Perhaps this might be different in larger series.

### DISCUSSION

To the best of our knowledge few studies have been reported on the prognostic implications of nuclear size in breast cancer. A lot of investigations have been carried out using the nuclear grading system proposed by Black & Speer (1957) or using other nuclear parameters such as nuclear crowding and lobulation (Stenkvist *et al.*, 1979). Their major drawbacks seem to be their lack of reproducibility and their subjectivity (Freedman *et al.*, 1979; Stenkvist *et al.*, 1979).

A more objective criterion, said to have prognostic implications, was proposed by Kallenberger *et al.* (1967) who correlated sex chromatin, DNA content and nuclear size with the evolution of breast cancer. Wallgren & Zajicek (1976) studied survival rates according to nuclear sizes on needle-aspirates of 359 breast tumours; increase in nuclear size was associated with decreased survival rate. Both 5-year and 10-year survival rates (93 and 82%) were higher among patients whose smears showed small ($<9{\cdot}5$ $\mu$m) or fairly small (9·5–12 $\mu$m) carcinoma nuclei. The lowest rates (67 and 58% respectively) were shown by those with large nuclei ($>19$ $\mu$m) (Wallgren & Zajicek, 1976). These data were confirmed by Zajdela *et al.* (1979) though they used different subgroups. Small nuclear types ($\leqslant 12$ $\mu$m) had a 5-year survival, free of disease, in 90% of cases *vs* only 58% for the large nuclear types ($>12$ $\mu$m).

All these studies have been carried out on smears of aspiration-biopsies, but our investigation used tissue sections. As a first step, we had to verify the comparability of measurements; a comparative study showed a shrinkage of breast cancer cells and nuclei after fixation and processing of tissue sections. Diameters on smears are about $1{\cdot}55\times$ larger than on tissue sections (van Bogaert & de Muylder, 1980). Therefore, we chose 8 and 12 $\mu$m as the lower and higher levels separating small and large nuclei on tissue sections; the 8$\mu$m level corresponded approximately to the value defining the cytological small nuclear type. The respective percentages of our cases, as well as the subdivision according to diameters, were closer to that reported by Wallgren *et al.* (1976) than to those of Zajdela *et al.* (1979). Accordingly, some correspondence does exist between cytological studies and histological ones. Further classification according to histological subtypes is more debatable, primarily because it may lead to unnecessary subdivisions; moreover, different histological criteria for typing and grading tumours may be used by various observers. Thus, the corrected values reported by Ashton *et al.* (1975) for duct and lobular carcinomas were respectively $7{\cdot}5 \pm 1{\cdot}8$ and $6{\cdot}3 \pm 0{\cdot}9$ $\mu$m; their mean nuclear sizes were distinctly lower than in our material ($10{\cdot}0 \pm 1{\cdot}5$ and $7{\cdot}5 \pm 1{\cdot}5$ $\mu$m).

Concerning prognostic implications, the sole convincing observation in our study was the relationship between increasing nuclear size and mortality from disseminated disease. Our finding is partly in keeping with Friedell's (1978) observation of a relationship between nuclear grade and nodal status, except for the less differentiated (large nuclei) tumours. The author saw a systematic relationship between recurrence and nuclear grade, which was not apparent in our study. Antoniades & Spector (1979) reported a correlation between estrogen-receptor (ER) values and cellularity, cell and nuclear sizes. $ER^+$ tumours had a mean nuclear diameter of 9·7 $\mu$m, $ER^-$ cancers had a much larger, 11·1 $\mu$m diameter. Silvestrini *et al.* (1979) found an inverse relationship between ER and proliferative activity; differentiated tumours had a low DNA-labelling index and high levels of ER. Finally, Hähnel *et al.* (1979) demonstrated that $ER^+$ cancers had a significantly better chance of survival, and delayed recurrence even in $N^+$ cases.

Concurrent morphological and functional data in the literature indicate a biological relationship between nuclear size, morphofunctional differentiation of breast tumours, and their invasiveness. In our preliminary findings there was a significant increase in mortality from disseminated breast cancer, which paralleled rising nuclear diameters. Other parameters of behaviour were less clearly influenced by this factor. The particular behaviour of large-nucleus tumours might be due to the small number of cases. Moreover, more than one discriminant is probably needed to identify more accurately the patients at risk of early recurrence.

The helpful assistance of Claudine Lahaye is gratefully acknowledged.

### REFERENCES

ANTONIADES, K. & SPECTOR, P. R. (1979) Correlation of estrogen receptor levels with histology and cytomorphology in human mammary cancer. *Am. J. Clin. Pathol.*, **71**, 497.

ASHTON, P. R., HOLLINGSWORTH, A. S. & JOHNSTON, W. W. (1975) The cytopathology of metastatic breast cancer. *Acta Cytol.*, **19**, 1.

BLACK, M. M. & SPEER, F. D. (1957) Nuclear structure in cancer tissue. *Surg. Gynec. Obstet.*, **105**, 907.

FREEDMAN, L. S., EDWARDS, D. N., MCCONNELL, E. M. & DOWNHAM, D. Y. (1979) Histological grade and other prognostic factors in relation to survival of patients with breast cancer. *Br. J. Cancer*, **40**, 44.

FRIEDELL, H. (1978) Identification of breast cancer patients with high risk of early recurrence after radical mastectomy. II. Clinical and pathological correlations. *Cancer*, **42**, 2809.

HÄHNEL, R., WOODINGS, T. & VIVIAN, A. B. (1979) Prognostic value of estrogen receptors in primary breast cancer. *Cancer*, **44**, 671.

KALLENBERGER, A., HAGMANN, A., MEIER-RUGE, W. & DESCOEUDRES, C. (1967) Beziehungen zwischen Sexchromatinvorkommen, Kerngrösse und ihr Bedeutung für die Ueberlebenszeit. *Schweiz. Med. Wochenschr.*, **97**, 678.

SAVINO, A. & KOSS, L. G. (1971) The evaluation of sex chromatin as a prognostic factor in carcinoma of the breast. A preliminary report. *Acta Cytol.*, **15**, 372.

SILVESTRINI, R., DAIDONE, M. G. & DI FRONZO, G. (1979) Relationship between proliferative activity and estrogen receptors in breast cancer. *Cancer*, **44**, 665.

STENKVIST, B., WESTMAN-NAESER, S., VEGELIUS, J. & 4 others (1979) Analysis of reproducibility of subjective grading systems for breast carcinoma. *J. Clin. Pathol.*, **32**, 979.

VAN BOGAERT, L. J. & MALDAGUE, P. (1978) Histological classification of pure primary epithelial breast cancer. *Hum. Pathol.*, **9**, 175.

VAN BOGAERT, L. J. & DE MUYLDER, C. (1980) Nuclear diameters of breast cancer cells in tissue section. *Anal. Quantit. Cytol. J.*, **2**, 55.

WALLGREN, A., SILFVERSWARD, C. & ZAJICEK, J. (1976) Evaluation of needle aspirates and tissue sections as prognostic factors in mammary carcinoma. *Acta Cytol.*, **20**, 313.

WALLGREN, A. & ZAJICEK, J. (1976) The prognostic value of the aspiration biopsy smear in mammary carcinoma. *Acta Cytol.*, **20**, 479.

ZAJDELA, A., SARAVIA DE LA RIVA, L. & GHOSSEIN, N. A. (1979) The relation of prognosis to the nuclear diameter of breast cancer cells obtained by cytologic aspiration. *Acta Cytol.*, **23**, 75.